# Three wise centromere functions: see no error, hear no break, speak no delay

**DOI:** 10.1038/embor.2013.181

**Published:** 2013-11-15

**Authors:** Tomoyuki U Tanaka, Lesley Clayton, Toyoaki Natsume

**Affiliations:** 1 Centre for Gene Regulation and Expression, College of Life Sciences, University of Dundee, Dow Street, Dundee DD1 5EH, UK; 2 Present address: Molecular Function Laboratory, Centre for Frontier Research, National Institute of Genetics, Mishima, Shizuoka, Japan

**Keywords:** centromere, kinetochore, microtubule attachment, sister-kinetochore bi-orientation, sister-chromatid cohesion, DNA replication timing

## Abstract

The main function of the centromere is to promote kinetochore assembly for spindle microtubule attachment. Two additional functions of the centromere, however, are becoming increasingly clear: facilitation of robust sister-chromatid cohesion at pericentromeres and advancement of replication of centromeric regions. The combination of these three centromere functions ensures correct chromosome segregation during mitosis. Here, we review the mechanisms of the kinetochore–microtubule interaction, focusing on sister-kinetochore bi-orientation (or chromosome bi-orientation). We also discuss the biological importance of robust pericentromeric cohesion and early centromere replication, as well as the mechanisms orchestrating these two functions at the microtubule attachment site.

See the Glossary for abbreviations used in this article.

## Introduction

**Figure 1:**
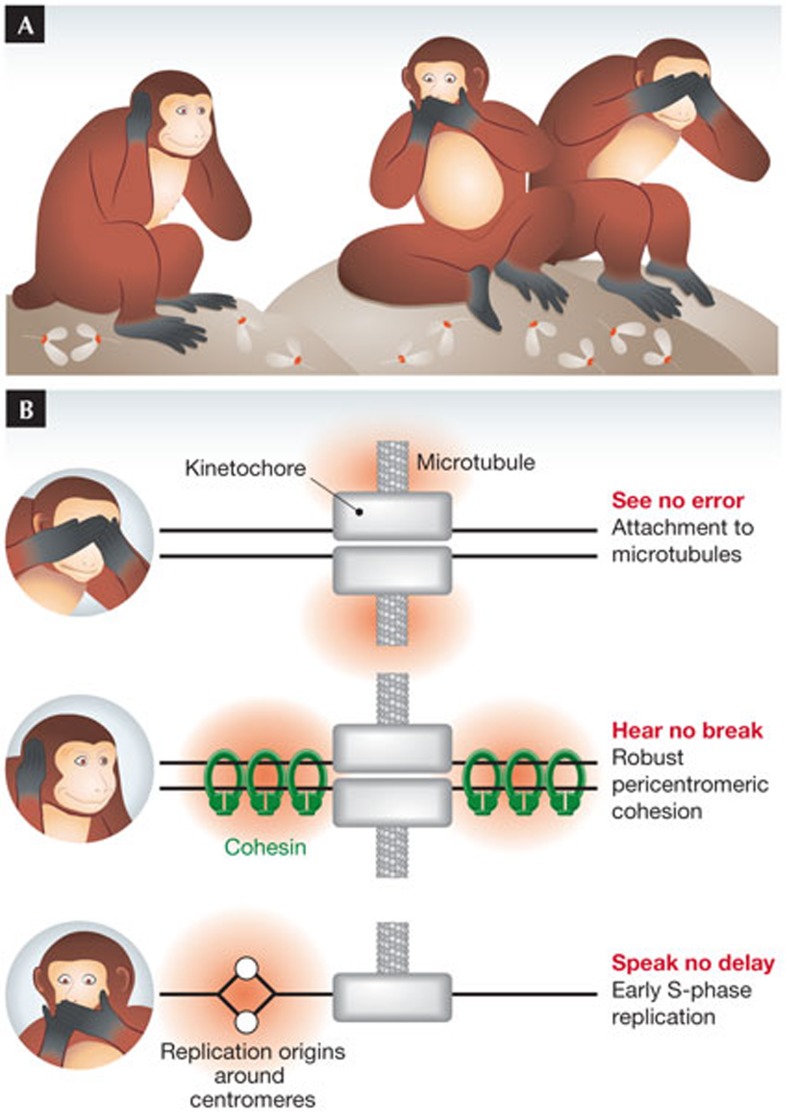
Three wise centromere functions. (**A**) The three wise monkeys, which are mystic apes known as *Mizaru*, *Kikazaru* and *Iwazaru* in Japanese. Together they symbolize the proverbial principle to see no evil, hear no evil, speak no evil. (**B**) The three wise functions of the centromere: orchestration of proper microtubule attachment (see no error), robust sister-chromatid cohesion (hear no break) and early S-phase replication (speak no delay) at the same chromosome site.

The centromere is a specialized chromosome site that has essential roles in chromosome segregation. To maintain genetic integrity, eukaryotic cells must segregate their chromosomes properly to opposite spindle poles before cell division, and the centromere is crucial to this process. It promotes the assembly of the multiprotein complex called the kinetochore that provides the major attachment site for spindle microtubules. This kinetochore–microtubule interaction powers the motion of chromosomes towards spindle poles to accomplish chromosome segregation in anaphase. This is, undoubtedly, the main function of the centromere. However, it is not the sole function; there are at least two additional features of the centromere that ensure high-fidelity chromosome segregation. In this Review, we discuss these three roles and compare them to the proverbial three wise monkeys, who are three mystic apes, known as *Mizaru*, *Kikazaru* and *Iwazaru* in Japanese (see no evil, hear no evil, speak no evil in English; Fig 1A). Similarly to the three wise monkeys, the centromere has three ‘wise’ functions, all involved in ensuring correct chromosome segregation, which we interpret as see no error, hear no break and speak no delay.

**Figure 2:**
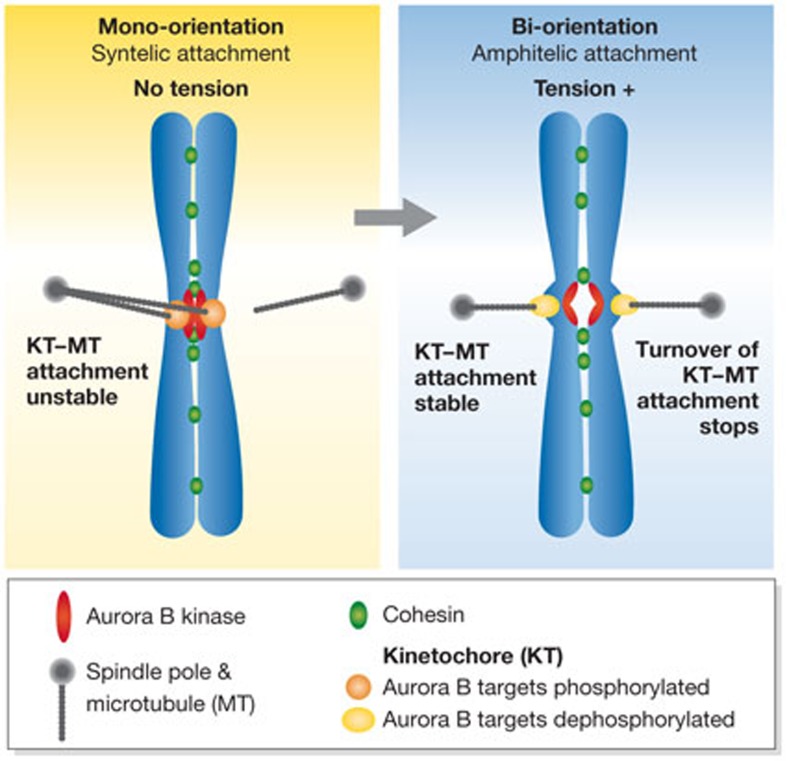
Error correction promoted by Aurora B kinase. Aurora B kinase promotes error correction, leading to sister-kinetochore bi-orientation. When tension is not applied on a kinetochore–microtubule attachment, kinetochore phosphorylation by Aurora B causes its turnover (left). According to the Aurora B spatial separation model, on bi-orientation, kinetochores delocalize from Aurora B, which causes kinetochore dephosphorylation and stops the turnover (right). On bi-orientation, kinetochore–microtubule attachment could be stabilized also due to its intrinsic properties.

Pericentromeres—the chromosomal regions around the centromeres—are associated with robust sister-chromatid cohesion (Fig 1B; [1,2]), which facilitates the attachment of sister kinetochores to microtubules from opposite spindle poles, a status known as sister-kinetochore bi-orientation or chromosome bi-orientation [3]. Sister-kinetochore bi-orientation is at the heart of the chromosome segregation mechanism and must be established before the onset of anaphase. Second, centromeric regions, which include core centromeres and pericentromeres, are replicated early during the S phase in many organisms, including several yeast species (Fig 1B). The early replication of centromeric DNA seems to be crucial for timely kinetochore assembly and microtubule attachment, at least in the budding yeast *Saccharomyces cerevisiae* [4]. Thus, in our analogy of the three wise monkeys, the centromere coordinates proper microtubule attachment (see no error), robust sister-chromatid cohesion (hear no break) and early S-phase replication (speak no delay) at the same chromosome site (Fig 1B).

The centromere in budding yeast is known as a point centromere because a small DNA region of about 130 bp suffices for its function [5,6]. Due to its small size, the centromere can be easily transferred to a new chromosome site and, remarkably, all the centromere features mentioned above are re-established [7,8,9]. This feature indicates that the centromere is sufficient to direct all these functions.

In this article, we focus on recent discoveries in two model eukaryotic organisms, the budding yeast *S. cerevisiae* and the fission yeast *Schizosaccharomyces pombe*, and extend our arguments to metazoan cells. For simplicity, we centre our discussion on mitosis and only briefly mention meiosis. Comprehensive reviews of chromosome segregation in meiosis are available [10,11].

## Centromeres promote interaction with spindle microtubules

The main role of the centromere is to promote the assembly of the kinetochore, which attaches the chromosome to spindle microtubules [12,13,14,15]. At the start of mitosis (prometaphase), the kinetochore interacts initially with the lateral surface of a single microtubule [16,17]. As the microtubule shrinks, its plus end eventually reaches the kinetochore, which is then tethered at the microtubule end—end-on attachment. Subsequently, its sister kinetochore establishes an end-on attachment with microtubules extending from the opposite spindle pole, thus establishing sister-kinetochore bi-orientation before the onset of anaphase [3,18]. The kinetochore–microtubule interaction is monitored by the spindle assembly checkpoint, which prevents sister-chromatid separation and anaphase onset until all chromosomes successfully establish bi-orientation [19,20,21]. These topics are not discussed extensively in this article. Instead, we discuss the error-correction mechanism that ensures sister-kinetochore bi-orientation and focuses on the role of Aurora B kinase in this process.

Error correction relies on the development of tension across sister kinetochores; when an aberrant attachment is made and no tension is applied, the kinetochore–microtubule attachment remains weak, and the connection is dissolved and reformed—that is, turned over [3,22]. If bi-orientation is established and tension is applied, turnover no longer occurs and kinetochore–microtubule attachment is stabilized. Thus, the error-correction mechanism removes aberrant attachments and promotes bi-orientation. A key regulator of this mechanism is Aurora B kinase, known as Ipl1 in budding yeast (Table 1; [3,23]), which forms the chromosome-passenger complex (CPC) together with INCENP, Survivin and Borealin (also called Dasra-B) [24]. The CPC was so named because it localizes at the inner centromere until anaphase onset, but then re-localizes to the central spindle during anaphase. Budding yeast Aurora B kinase promotes the turnover of kinetochore–microtubule attachment when there is no tension, for example on syntelic attachment, in which both sister kinetochores attach to microtubules from the same spindle pole (Fig 2; [25,26]). Consistent with this finding, aberrant kinetochore–microtubule attachments accumulate in mammalian cells when the Aurora B kinase is defective [27,28]. The function of Aurora B is dependent on its phosphorylation of several kinetochore components that form the kinetochore–microtubule interface [23].

How, then, is kinetochore–microtubule attachment stabilized once bi-orientation is established and tension is applied (Fig 2)? In budding yeast, sister kinetochores were suggested to be pulled in opposite directions on establishment of bi-orientation, moving kinetochores away from Aurora B-localizing sites [25]. This leads to the dephosphorylation of kinetochore components, which stabilizes the kinetochore–microtubule attachment, and is known as the Aurora B spatial separation model. This model has been supported by the properties of INCENP mutants and by the Aurora B localization pattern in budding yeast [25,29,30]. Further strengthening of this model also comes from results in budding yeast and mammalian cells, as Aurora-B-dependent phosphorylation of kinetochore components is reduced when tension is applied [31,32,33]. In addition, ectopic targeting of Aurora B to the outer kinetochores destabilizes kinetochore–microtubule attachment during metaphase in mammalian cells [32]. Relevant to this model, when the kinetochore becomes less phosphorylated, it recruits more protein phosphatase 1, thus establishing a positive feedback loop that promotes kinetochore dephosphorylation [34]. The Aurora B spatial separation model also explains why the CPC, which contains Aurora B, re-localizes to the spindle mid-zone during anaphase [24]. If this were not the case, Aurora B would localize with kinetochores again during anaphase, when tension is substantially reduced, which would once more destabilize kinetochore–microtubule attachment [35,36,37].

The Aurora B spatial separation model, however, has been challenged by the findings of two recent studies in budding yeast. In the first study, kinetochores were purified from yeast cells and their microtubule attachment was studied *in vitro* [38,39]. Optical tweezers were used to apply varying amounts of tension on the attachment. Intriguingly, although Aurora B was absent from the purified kinetochores, kinetochore–microtubule interaction became more stable with increasing tension [38]. This finding raises the possibility that tension stabilizes kinetochore–microtubule interaction independently of Aurora B spatial separation from kinetochores. The kinetochore detachment from a microtubule observed in this system was accompanied by microtubule depolymerization, the rate of which was enhanced when tension was reduced [38,39]. Whether depolymerization happens during error correction *in vivo* will be crucial to determine. In any case, tension could stabilize kinetochore–microtubule interaction both by reductions in kinetochore phosphorylation, which is dependent on Aurora B spatial separation from kinetochores, and through intrinsic properties of the kinetochore–microtubule interaction, which do not require Aurora B function [38].

The second study characterized mutants of INCENP—Sli15 in budding yeast—that cannot interact with Survivin—Bir1 in yeast [40]. In such mutants, Survivin could no longer target INCENP–Aurora B to the centromere and became dispensable for cell viability. Nevertheless, how can cells undergo error correction to establish bi-orientation without Survivin? One possibility is that there is a Survivin-independent mechanism for targeting CPC to the centromere, where Aurora B still promotes error correction in the absence of Survivin. An alternative, more radical, possibility is that as long as Aurora B is activated its localization at the centromere might not be essential for error correction. In such a scenario, the INCENP mutants could cause premature spindle localization of the CPC, leading to activation of Aurora B on the spindle instead of at the centromere [40]. Nevertheless, localization of Aurora B at the centromere could still enhance the fidelity of error correction—even if it is not essential for it—and, if Aurora B is at the centromere, kinetochores could have to delocalize from it when bi-orientation is established. Both possibilities are interesting and, whichever is true, it would point to a novel regulation of Aurora B.

In summary, Aurora B kinase has a key role in promoting the turnover of kinetochore–microtubule interactions for error correction, leading to sister-kinetochore bi-orientation. How the kinetochore–microtubule interaction is stabilized when bi-orientation is established and tension is applied is still a matter of debate (Sidebar A). Aurora B spatial separation from the kinetochore has been a popular explanation. However, more intrinsic properties of kinetochore–microtubule interaction might also be involved, and whether Aurora B localization at the centromere is essential for bi-orientation remains unclear, at least in budding yeast.

## Centromeres facilitate robust pericentromeric cohesion

Sister-chromatid cohesion relies on cohesins Scc1 (also called Mcd1 or Rad21; Table 1), Scc3, Smc1 and Smc3, which form a tetrameric, ring-shaped complex that embraces the sister chromatids [41,42]. Cohesins are loaded onto chromatin in telophase in fission yeast and mammalian cells, and in G1 phase in budding yeast. This process is facilitated by the cohesin loader complex—Scc2–Scc4 in budding yeast—before sister chromatids are linked during DNA replication. The establishment of this linkage is coupled with Smc3 acetylation, catalysed by the Eco1 acetylase (also known as Ctf7) during S phase, which counteracts the activity of Wapl (also called Wpl1 and Rad61), which facilitates cohesin dissociation from chromosomes [43,44,45,46]. In budding yeast, cohesins are distributed along chromosome arms at distinct sites, preferentially in intergenic regions between convergent genes, but show particularly high enrichment in the 20–50 kb surrounding centromeres [8,47,48,49,50]. As discussed below, the cohesins enriched at pericentromeric regions are crucial to the establishment of sister-kinetochore bi-orientation and, therefore, to ensure proper chromosome segregation.

How are cohesins enriched in the region around centromeres in budding yeast? The yeast point centromere has consensus sequences *CDEI*, *CDEII* and *CDEIII* that span only 130 bp, and is called a point centromere [5,6]. Pioneering studies that used minichromosomes and centromere translocation on a chromosome have demonstrated that the point centromere is necessary and sufficient for both recruitment of a high density of cohesin to pericentromeric regions and the resulting robust cohesion [8,51,52]. What feature(s) of the point centromere allows the recruitment of cohesins? The centromere promotes kinetochore assembly and distinct kinetochore components seem to have important roles in this process [8,53]. In fact, the Ctf19 kinetochore complex (also called COMA) is important for the recruitment of the Scc2–Scc4 complex to the centromere, which in turn promotes cohesin enrichment at pericentromeric regions [54,55,56].

**Figure 3:**
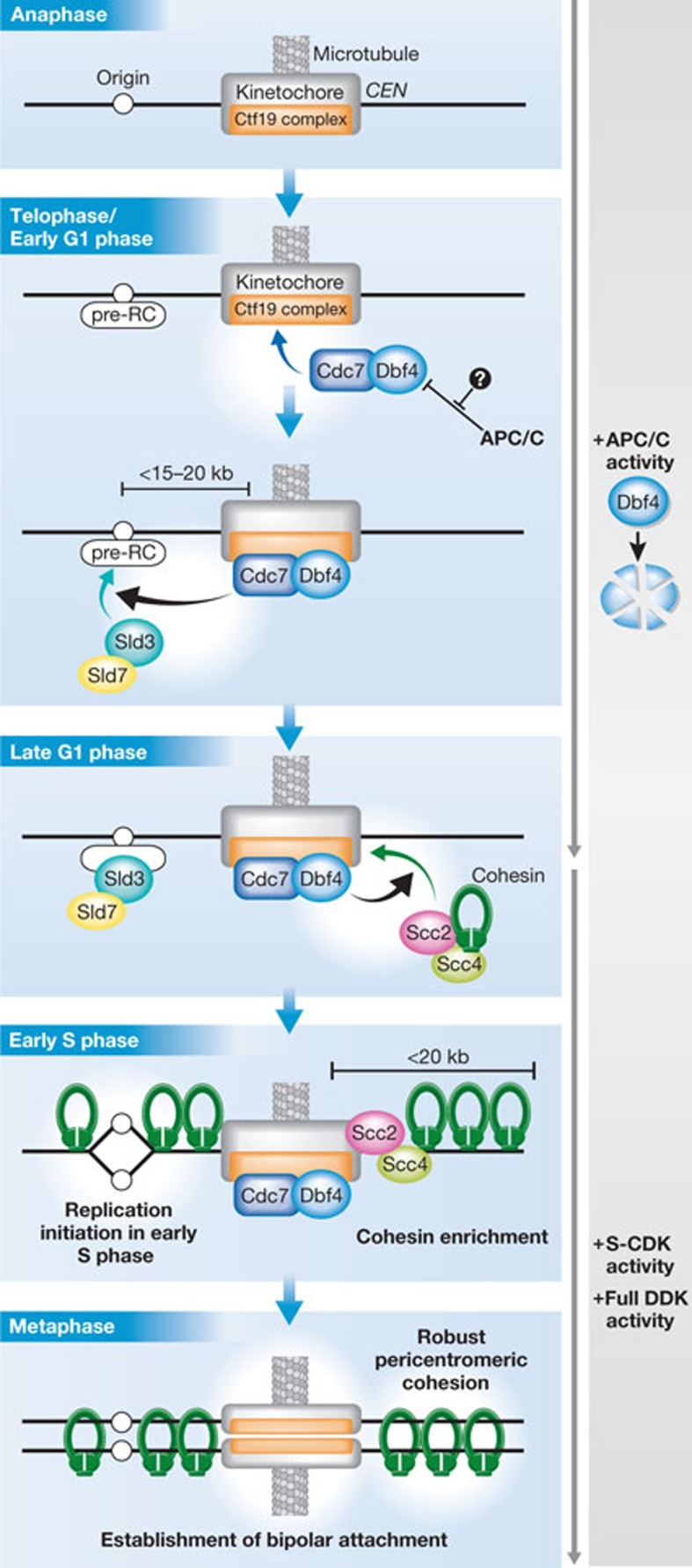
Roles of DDK at kinetochores in budding yeast. DDK (Dbf4–Cdc7) promotes pericentromeric sister-chromatid cohesion and advances the replication of centromeric regions in budding yeast [57]. DDK is recruited to kinetochores during telophase to early G1 phase by the Ctf19 kinetochore complex. The DDK at kinetochores in turn recruits Sld3–Sld7 replication initiation proteins to pericentromeric replication origins in telophase to early G1 phase, as well as the Scc2–Scc4 cohesin loader to centromeres in the late G1 phase. pre-RC, pre-replicative complex.

**Figure 4:**
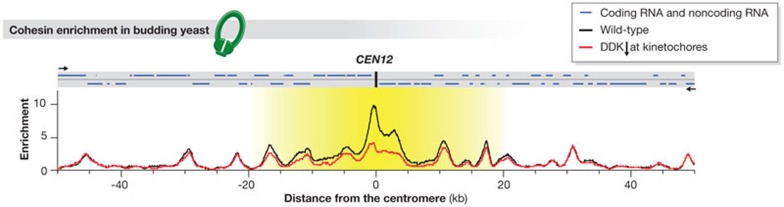
Cohesin distribution at pericentromeres in budding yeast. DDK at kinetochores enhances the amount of cohesins at centromeres and at pericentromeric regions (up to 20 kb from centromeres) in budding yeast [57]. The graph shows the amount of Scc1 along the indicated chromosome region around the centromere of chromosome XII (*CEN12*). The Scc1 amount was measured by chromatin immunoprecipitation, followed by high-throughput DNA sequencing (ChIP-seq) [149,150], in *DBF4*+ (no tag; black line) and *DBF4-myc* (in which the amount of DDK is reduced at kinetochores; red line) cells. Adapted from Natsume T *et al* (2013) *Mol Cell* **50:** 661–674 [57].

A recent study identified an effector of this process in budding yeast: the Ctf19 complex recruits the Dbf4–Cdc7 kinase (Dbf4-dependent kinase; DDK) to the kinetochore during telophase to early G1 phase (Fig 3; [57]). Intriguingly, the majority of Dbf4 is targeted for degradation by the APC/C—with adaptor Cdh1—during this phase [58,59], but Dbf4 at kinetochores evades this degradation by an unknown mechanism. DDK at the kinetochore promotes the loading of Scc2–Scc4 to the centromere, which leads to enrichment of cohesin at pericentromeres (Fig 4). This process requires Cdc7 kinase activity, but the relevant substrates are unknown. Phosphorylation of Scc2–Scc4 might enhance its affinity for centromeres, or phosphorylation of the Ctf19 complex could mark a landing pad for Scc2–Scc4. Notably, DDK loading on the kinetochore in telophase to early G1 phase is required, but is not sufficient, for Scc2–Scc4 recruitment to the centromere [57]. Recruitment requires assembly of the cohesin ring, which is completed by Scc1 expression in the late G1 phase [60].

Once Scc2–Scc4 and cohesins are loaded onto the centromere in late G1 phase, the cohesin ring embraces the chromosomes. This process requires the ATPase activity of the Smc1 and Smc3 heads [61], which are thought to interact with the Smc1–Smc3 hinge, where they open the cohesin ring to trap the chromosomal DNA inside [62]. On DNA replication, cohesin rings embrace both sister chromatids, which establishes sister-chromatid cohesion. In budding yeast, the S phase is followed immediately—with no G2 phase—by the establishment of a bipolar spindle, which promotes sister-kinetochore bi-orientation [4,63]. On bi-orientation, kinetochore-attached microtubules pull sister centromeres apart, leading to sister-chromatid separation up to 10 kb around the centromere before the onset of anaphase [64,65,66,67]. This pericentromeric sister-chromatid separation seems to cause translocation of cohesin rings from the centromere to the pericentromeric regions [54,68]. Accordingly, the cohesins at pericentromeric regions maintain robust sister-chromatid cohesion until anaphase onset, when separase cleaves Scc1 and opens the cohesin rings, triggering sister-chromatid separation and segregation [69,70]. By contrast, cohesins loaded at the centromere after DNA replication—and, therefore, not embracing both sister chromatids—seems to be less mobile and remain in the vicinity of the centromere [68]. Nevertheless, how cohesins interact topologically with centromeric chromatin is still a topic of debate [71].

The Ctf19 kinetochore complex has orthologues in fission yeast and in metazoan cells, known, respectively, as the Sim4 complex and CCAN [13,72]. Whether these orthologues are involved in enriching cohesins at pericentromeres, as they are in budding yeast, remains unknown. In fission yeast, however, pericentromeric heterochromatin has an important role in enriching cohesins. The mechanisms of pericentromeric heterochromatin formation in fission yeast have been reviewed [73,74,75]. Briefly, small RNAs transcribed from pericentromeric regions (known as outer repeats) are processed by the RNA interference (RNAi) pathway, which brings the methyltransferase Clr4 to this region and promotes methylation of histone H3 at Lys 9 (H3K9m). H3K9m is recognized and bound by Swi6, which organizes heterochromatin. Heterochromatin is self-sustaining because H3K9m further activates the RNAi pathway. Notably, fission yeast pericentromeric heterochromatin facilitates cohesin accumulation, and the interaction between Swi6 with Psc3 (an orthologue of budding yeast Scc3) has an important role in this context [76,77,78]. A high density of cohesins at pericentromeres leads to robust sister-chromatid cohesion. In vertebrate cells, it is still unclear whether RNAi is involved in heterochromatin formation, but relevant RNA-mediated chromatin modifications have been suggested [74].

**Figure 5:**
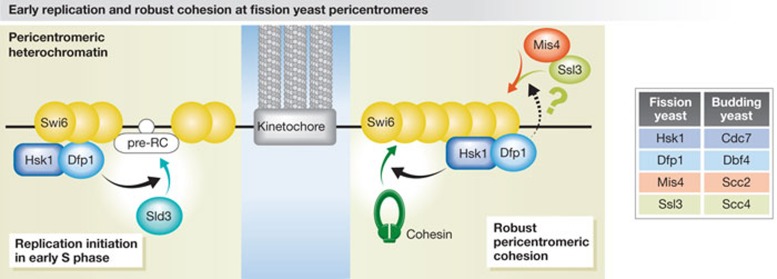
Roles of DDK at fission yeast pericentromeric heterochromatin. In fission yeast, the heterochromatin protein Swi6 recruits DDK (called Dfp1–Hsk1) to pericentromeres [79]. Dfp1–Hsk1 in turn promotes the recruitment of cohesins and the replication initiation protein Sld3 to pericentromeric regions, leading to robust sister-chromatid cohesion and early S-phase replication of these regions [79,135]. The table on the right shows orthologues in fission and budding yeast.

Interestingly, fission yeast DDK is recruited to heterochromatin by Swi6 and has an important role in cohesin enrichment and robust cohesion (Fig 5) [79]. By contrast, budding yeast has no Swi6 orthologue or canonical heterochromatin. Rather DDK is recruited by kinetochore components [57], as discussed above. Notably, loading of Scc2 and cohesins to chromosomes in *Xenopus* egg extracts also requires DDK [80] and the pre-replicative complex [81,82], which therefore seems to be a process active at replication origins rather than at centromeric regions. In summary, the roles of DDK in cohesin recruitment seem to be conserved among organisms, but DDK recruitment to chromosomes occurs in different contexts: in a kinetochore-dependent manner in budding yeast, to heterochromatin in fission yeast, and in a pre-replicative complex-dependent manner in *Xenopus*. In budding and fission yeast, DDK recruitment to the kinetochore and pericentromeric heterochromatin leads to cohesin enrichment at pericentromeres. Intriguingly, in both yeast species, DDK is also involved in advancing replication timing at centromeric regions, which is the focus of the next section.

In vertebrate cells, as in fission yeast, HP1 (an orthologue of fission yeast Swi6) binds to H3K9m, leading to heterochromatin formation [83]. In contrast to fission yeast, however, there is no evidence that the H3K9m–HP1 pathway and DDK are involved in cohesin enrichment at pericentromeric heterochromatin [84,85]. Yet, in vertebrate cells, cohesins are removed from chromosome arms in prophase [86,87] and must be protected at centromeric regions in the transition from prophase to metaphase. Cohesin removal from chromosome arms is dependent on phosphorylation of SA1 and SA2 (orthologues of *S. cerevisiae* Scc3), which is catalysed by Aurora B and Plk1 kinases, and is known as the prophase pathway. Shugoshin has a key role in protecting centromeric cohesins from the prophase pathway [88] by recruitment of phosphatase 2A (PP2A), which reverses SA1/SA2 phosphorylation [89,90]. Cohesin removal from chromosome arms is also facilitated by Wapl in prophase [91,92]. This is triggered by phosphorylation of Sororin, which abrogates its Wapl-counteracting function [93]. At centromeres, however, Sororin is dephosphorylated by Shugoshin–PP2A, and this contributes to protection of cohesins from prophase to metaphase [94,95].

Shugoshins are also found at the centromere during mitosis in budding and fission yeast, but here their role is not to protect cohesins but rather to achieve high-fidelity sister-kinetochore bi-orientation, probably by assistance of Aurora B function [96]. Nevertheless, during meiosis I, Shugoshin does protect cohesins at the centromere in yeast and vertebrates by recruitment of PP2A to the centromere [89,97,98], similarly to what happens in vertebrate mitosis. However, in meiosis I, Shugoshin–PP2A targets and protects Rec8 (meiotic paralogue of Scc1) from separase-dependent cleavage, rather than protecting Scc3 from the prophase pathway, as occurs in vertebrate mitosis. Thus, the role of Shugoshin–PP2A in protecting cohesins at centromeric regions is conserved in evolution, although the mechanism is different in the contexts of mitosis and meiosis.

Robust sister-chromatid cohesion at pericentromeres is crucial for high-fidelity chromosome segregation in organisms from yeast to humans. For example, insertion of ectopic sequences into pericentromeres reduces the levels of cohesins, leading to frequent chromosome loss in budding yeast [54]. Several pieces of evidence suggest that pericentromeric cohesion facilitates sister-kinetochore bi-orientation, which is essential for chromosome segregation and must be established before anaphase onset. For example, if Scc1 is depleted in budding yeast, both sister centromeres often attach to microtubules from the same pole and, therefore, bi-orientation fails [66]. In addition, a specific reduction of pericentromeric cohesin leads to frequent failure in bi-orientation, and rescue of cohesion alleviates such bi-orientation defects [56].

Sister-kinetochore bi-orientation could be achieved by two kinds of mechanism: kinetochore geometry and tension-dependent error correction [3,99]. Aberrant kinetochore–microtubule attachment, such as syntelic attachment, might be avoided by reliance on the back-to-back geometry of sister kinetochores. When one kinetochore attaches to a microtubule, constraints in its geometry make its sister kinetochore face the opposite direction, which allows attachment only to a microtubule from the opposite pole. However, once an aberrant attachment is made, kinetochore geometry cannot correct it. Therefore, a second, error-correction mechanism is necessary. This error correction relies on differential stability of the kinetochore–microtubule interaction in the presence and absence of tension across sister kinetochores. Both kinetochore geometry and tension-dependent error correction could be facilitated by sister-chromatid cohesion at centromeric regions [8]. For example, sister-kinetochore geometry could be organized by robust cohesion at pericentromeres rather than at core centromeres, as found in fission yeast [100]. There is also evidence that kinetochore geometry is present in budding yeast [101], in which the point centromere is looped out from the pericentromere [71,102], and this configuration might contribute to kinetochore geometry. In addition, error correction would require centromeric cohesion. In budding yeast, tension across the two centromeres is sufficient for efficient bi-orientation of a non-replicated circular minichromosome carrying two centromeres [26]. This ability suggests that, in the context of authentic chromosomes, tension across sister centromeres should suffice for bi-orientation through error correction without the need to invoke kinetochore geometry. Such tension would be dependent on cohesin-dependent sister-chromatid cohesion at centromeric regions.

In budding yeast, only a single microtubule attaches to each kinetochore [103], whereas, in fission yeast and metazoan cells, there are multiple microtubules per kinetochore. In the latter case, an additional type of error is possible—that is, a single kinetochore could attach to microtubules from both spindle poles, which is called merotelic attachment. Such merotelic attachments could be discouraged by kinetochore geometry but could also be excluded through error correction [104]. Cohesion at centromeric regions could be important for both prevention and correction of merotelic attachments. Indeed, when cohesion is weakened at centromeric regions, merotelic attachment is formed frequently in fission yeast [76,105,106] and in mammalian cells [107,108].

In summary, eukaryotic cells accumulate cohesins at centromeric regions to establish robust sister-chromatid cohesion. Budding yeast, fission yeast and vertebrates use different mechanisms to accumulate or protect cohesins at centromeric regions. Nevertheless, DDK and Shugoshin–PP2A have some common roles in these organisms, albeit in different contexts. Understanding how the roles of these factors have developed during evolution will be interesting (Sidebar A). In all these organisms, robust cohesion at centromeric regions is important to establish sister-kinetochore bi-orientation before anaphase onset, which is essential for proper chromosome segregation during the subsequent anaphase.

## The centromere advances its replication timing

In eukaryotic cells, the duplication of chromosomal DNA is a temporally regulated process and, crucially, the replication timing of a chromosome region is linked closely to its biological functions (see below). How, then, is DNA replication regulated temporally? DNA replication is initiated from multiple replication origins on a chromosome, in a process often termed origin firing. Although initiation of replication is a stochastic process at each origin, its average timing is under temporal regulation—that is, some origins tend to fire early and others late during S phase [109,110]. The mechanisms of such temporal regulation in budding yeast, fission yeast and metazoan cells have been reviewed [111,112]. For example, the roles of the histone deacetylase Rpd3 [113,114], forkhead box transcription factors Fkh1 and Fkh2 [115] and the telomere-binding protein Rif1 [116,117,118] in the programme of genome-wide replication timing have been identified. Intriguingly, the timing of initiation of replication is set at each origin in telophase to early G1 phase in *S. cerevisiae* and mammalian cells [119,120]. For example, in the early G1 phase of budding yeast, some early-replicating but not late-replicating origins are loaded with DDK, Sld3–Sld7 and Cdc45, all of which are required for replication initiation at licensed origins—origins with pre-replicative complex—in the subsequent S phase [121,122,123,124,125,126].

Importantly, DNA replication at centromeric regions is under distinct temporal regulation. Indeed, centromeric regions are replicated early in the S phase on all chromosomes of *S. cerevisiae* and other *Saccharomyces* species [127,128,129]. Centromeric regions in other yeast species, such as *Candida albicans* and *S. pombe*, and those in the protozoan parasite *Trypanosoma brucei* are also replicated early in the S phase [130,131,132]. Thus, replication of centromeric regions early in the S phase is a conserved feature in many yeast and protozoan species. Notably, the formation of a neocentromere in *C. albicans* advances the replication timing of its chromosomal site [131], which suggests that the presence of a centromere *per se* changes the timing of replication. Consistent with this finding, the point centromere of *S. cerevisiae* is sufficient to advance the initiation of replication in its neighbouring replication origins. Indeed, when the point centromere is transferred to another chromosome locus, a late-S-phase firing replication origin close to the new centromere site becomes an early-S-phase firing origin [9]. *S. pombe* pericentromeric replication origins are embedded within heterochromatin, which generally replicates in the late S phase in metazoan cells [133]. However, *S. pombe* pericentromeric origins show early-S-phase replication despite their location [130,131].

**Figure 6:**
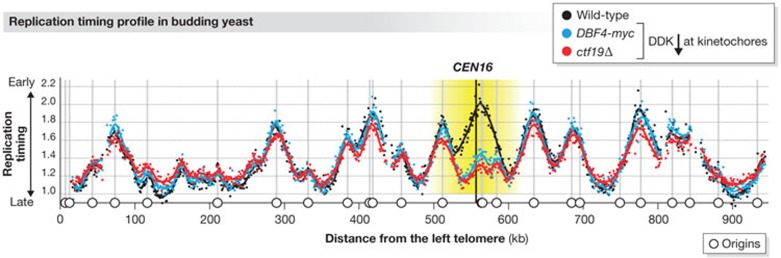
Profile of replication timing of a budding yeast chromosome. DDK at kinetochores advances replication timing of centromeric regions in budding yeast [57]. The graph shows the profile of replication timing of chromosome XVI. The profile was obtained from high-throughput DNA sequence reads [129] in S phase of wild-type (black dots and line), *Dbf4-myc* (blue) and *ctf19*Δ (red) cells. In *Dbf4-myc* and *ctf19*Δ cells, the amount of DDK at kinetochores is reduced. Adapted from Natsume T *et al* (2013) *Mol Cell* **50:** 661–674 [57].

To advance the replication timing of the centromeric region, in budding yeast, DDK is recruited to the kinetochore by the Ctf19 kinetochore complex in telophase to early G1 phase [57]. In turn the association of the Sld3–Sld7 complex—and probably other replication initiation proteins—with licensed replication origins within 15–20 kb from the centromeres is facilitated, which leads to firing of these origins in the early S phase (Fig 3). Indeed, if DDK is removed from the kinetochore, but not from replication origins on chromosome arms, replication is delayed at the centromeric region and not along the chromosome arms (Fig 6; [57]). Although the effect of DDK in advancing pericentromeric origin firing requires its kinase activity, the DDK substrates for this effect are unknown. However, DDK phosphorylates several subunits of the Mcm2–7 complex—a replicative helicase core and pre-replicative component—at each origin, and these are major substrates of DDK in the initiation of replication [134]. One possibility is that DDK at kinetochores advances replication initiation timing at pericentromeric origins by phosphorylating the same Mcm2–7 subunits at the same sites before the S phase. Consistent with this possibility, the *mcm5/bob1-1* mutation, which bypasses the essential role of DDK in initiation of DNA replication—phosphorylation of Mcm2–7 subunits—also enables otherwise late-firing origins to initiate replication early in the S phase [124]. Alternatively, other pre-replicative components, Sld3–Sld7, or other replication-initiation proteins, such as Cdc45, might be the relevant targets of kinetochore DDK for advancing replication timing at pericentromeres.

The same Ctf19 complex–DDK pathway, discussed above, is used for robust pericentromeric cohesion [57]. Although the replication timing and cohesion functions of DDK at the kinetochore both require its kinase activity, they are independent of each other. That is, the situations in which one function is lost and the other still effective can be engineered [57]. This independence suggests that the two functions rely on phosphorylation of different DDK substrates. Intriguingly, fission yeast DDK is also recruited to centromeric regions to facilitate robust cohesion and to advance replication timing (Fig 5; [79,135]). Nonetheless, the mechanism for DDK recruitment is different in budding yeast and fission yeast, because in fission yeast the heterochromatin protein Swi6 binds to and recruits DDK to pericentromeric regions [79,135].

In metazoan cells, although pericentromeric heterochromatin replicates late in the S phase [133], the timing of replication of the core centromere is a topic of debate. For example, one report suggested that *Drosophila* cells show early-S-phase replication at core centromeres that are associated with a centromere-specific histone H3 variant CENP-A [136], but another study concluded that the core centromere replicates during the mid-to-late S phase in this organism [137]. In mouse cells, the core centromere replicates earlier than the surrounding heterochromatin [138] and, consistently, when neocentromeres are formed in human cells, the CENP-A-binding core centromere replicates earlier than surrounding sequences [139]. Core centromeres, however, do not show this property when neocentromeres are formed in chicken DT40 cells [140]. Thus, in metazoan cells, the situation might differ depending on the organism and context.

Are there any advantages to replicating the centromere early in S phase? We suggest four possibilities. First, early-S-phase centromere replication has been proposed to be important for centromere identity, especially for the deposition of CENP-A, which is an epigenetic marker of the centromere [141,142,143]. This theory is based on the centromere replication early in S phase in *Drosophila* and *C. albicans* [131,136], but remains controversial as a report has suggested that the core centromere replicates during mid-to-late S phase in *Drosophila* [137]. Moreover, the timing of centromere replication is unlikely to influence CENP-A deposition in human cells, which seems to occur in the G1 phase rather than during S phase [144]. Second, early-S-phase centromere replication could allow an early assembly of the kinetochore, providing more time to establish correct kinetochore–microtubule interactions. This possibility has been proposed for budding yeast and, indeed, a delay in centromere replication in this organism increases the importance of the spindle assembly checkpoint for high-fidelity chromosome transmission [57]. This possibility might be pertinent in organisms, such as budding yeast, in which kinetochore assembly and microtubule attachment occur soon after centromere DNA replication [4]. Third, centromere replication early in S phase might be important for robust sister-chromatid cohesion when cellular growth is slowed. The turnover of cohesins is fast on chromosomes until they become engaged in sister-chromatid cohesion [145,146]. Thus, after cohesins are loaded on chromosomes in the late G1 phase, they might be lost from chromosomes if replication—and therefore cohesins' engagement in cohesion—does not happen quickly. This would explain why a common regulator, such as DDK, coordinates cohesin recruitment and early-S-phase replication at pericentromeres in budding and fission yeast [57,79,135]. Fourth, the repair of DNA damage might be more efficient in early- than in late-replicating regions [147]. For example, early replication correlates with a low rate of genetic mutations [147,148]. The centromere could be susceptible to DNA damage owing to a replication barrier—because of the presence of the kinetochore—or tension—generated by microtubule attachment—and, therefore, would require efficient DNA repair. Such repair could help to maintain the consensus sequence of a point centromere (in budding yeast) or the repetitive DNA sequence of a ‘regional’ centromere (in fission yeast and metazoan cells). These four possibilities are not mutually exclusive. Further studies will be necessary to test them.

In summary, in many yeast and protozoan species, the centromere DNA replicates early in the S phase. In budding and fission yeast, this regulation relies on DDK recruitment to the kinetochore and pericentromeric heterochromatin, respectively, which advances the initiation of replication at pericentromeric origins. At least in some metazoan cells, the core centromere replicates earlier than surrounding pericentromeric heterochromatin. However, the advantages of early-S-phase centromere replication are still a topic of debate (Sidebar A). At least in budding yeast, it is probably important for timely kinetochore assembly and for the efficient establishment of correct kinetochore–microtubule interactions.

## Conclusions and perspectives

The main function of the centromere is to promote kinetochore assembly for microtubule attachment. This attachment provides the major force that drives chromosome segregation and, therefore, must be established efficiently and correctly. For example, kinetochores need to establish the initial microtubule interaction efficiently in early mitosis, and subsequent sister-kinetochore bi-orientation must be correctly formed before chromosome segregation. However, kinetochore assembly is not the only centromere function. Centromeres also facilitate robust sister-chromatid cohesion at pericentromeres and promote early-S-phase replication of the centromeric regions. These two functions seem to help the main centromere function. Indeed, pericentromeric cohesion facilitates sister-kinetochore bi-orientation in yeast and metazoan cells. In addition, centromere replication early in S phase might allow timely kinetochore assembly for efficient microtubule interaction, at least in budding yeast. The exact mechanism for robust pericentromeric cohesion that promotes bi-orientation and the advantages of early centromere replication in various organisms remain to be addressed (Sidebar A). The evolutionary conservation of these centromere functions will be an important area of future research (Sidebar A).

Intriguingly, in budding and fission yeast, the two additional centromere functions are facilitated by a common regulator DDK, but are regulated independently of each other. To understand the molecular mechanisms involved, it will be crucial to identify the targets of DDK phosphorylation that are important in mediating these processes (Sidebar A). To determine the conservation of the DDK-dependent mechanisms in metazoan cells will also be interesting. Three wise functions of the centromere contribute greatly to correct chromosome segregation and we should attempt to understand in more detail how the centromere orchestrates all three functions at the same chromosome site.

## Figures and Tables

**Table 1:** Orthologues of proteins in yeasts and humans

Budding yeast	Fission yeast	Human
*DNA replication*		
DDK		
Cdc7	Hsk1	Cdc7
Dbf4	Dfp1/Him1/Rad35	Dbf4/Ask, Drf1
Sld3	Sld3	Treslin
Sld7	N.F.	N.F.
Cdc45	Cdc45/Sna41/Goa1	Cdc45
Mcm2–7		
Mcm5/Cdc46/Bob1	Mcm5/Nda4	Mcm5
*Sister-chromatid cohesion*		
Cohesin		
Scc1/Mcd1	Rad21	Scc1/Rad21
Rec8	Rec8	Rec8
Scc3/Irr1	Psc3	SA1, SA2
Smc1	Psm1	Smc1A, Smc1B
Smc3	Psm3	Smc3
Cohesin loader		
Scc2	Mis4	Nipbl
Scc4	Ssl3	KIAA0892/Mau2
Eco1/Ctf7	Eso1	Esco1, Esco2
Rad61/Wpl1	Wpl1	Wapl
N.F.	N.F.	Sororin/CdcA5
Cdc5	Plo1	Plk1
*Kinetochore/centromere*		
Cse4	Cnp1	CENP-A
Ctf19 complex/COMA	Sim4 complex	CCAN
Ctf19	Fta2	CENP-P
Mcm21	Mal2	CENP-O
Ctf3	Mis6	CENP-I
Chl4	Mis15	CENP-N
Sgo1	Sgo1, Sgo2	Shugoshin
PP1	PP1	PP1
PP2A	PP2A	PP2A
*Chromosome passenger complex*		
Ipl1	Ark1	Aurora B
Sli15	Pic1	INCENP
Bir1	Bir1/Cut17	Survivin
Nbl1	Nbl1	Borealin/Dasra-B
*Cell-cycle regulation*		
APC/C		
Cdh1	Ste9	Cdh1
*Chromatin regulation*		
N.F.	Clr4	Suv39h1
N.F.	Swi6	HP1
Rpd3	Clr6	HDAC1/2
Fkh1, Fkh2	Fhl1, Fkh2	Fox
Rif1	Rif1	Rif1

The following names are also used in the text as generic names across organisms: Scc1, Scc3, Smc1, Smc3, Eco1, Wapl, CENP-A, Shogoshin, Aurora B, INCENP and Survivin. N.F., no orthologue found or annotated in this organism.

**Figure illus1:**
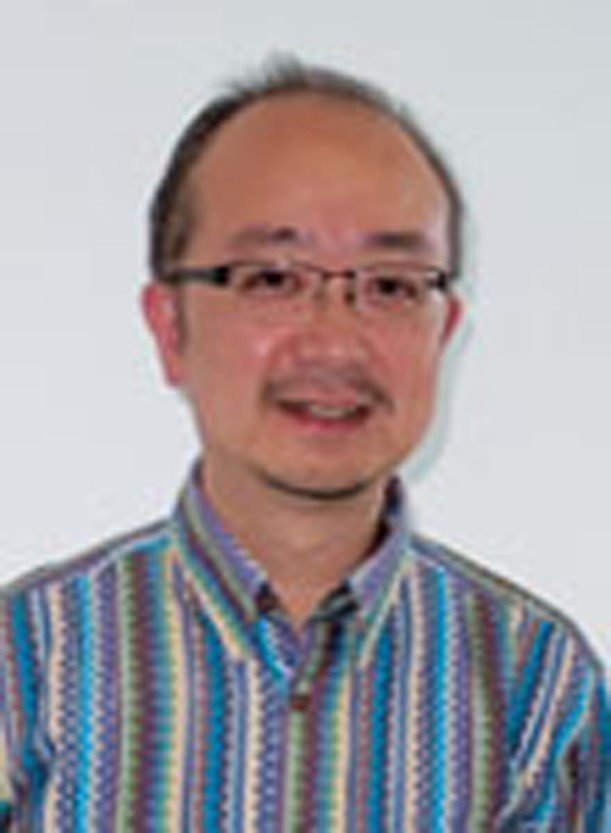
Tomoyuki U Tanaka

**Figure illus2:**
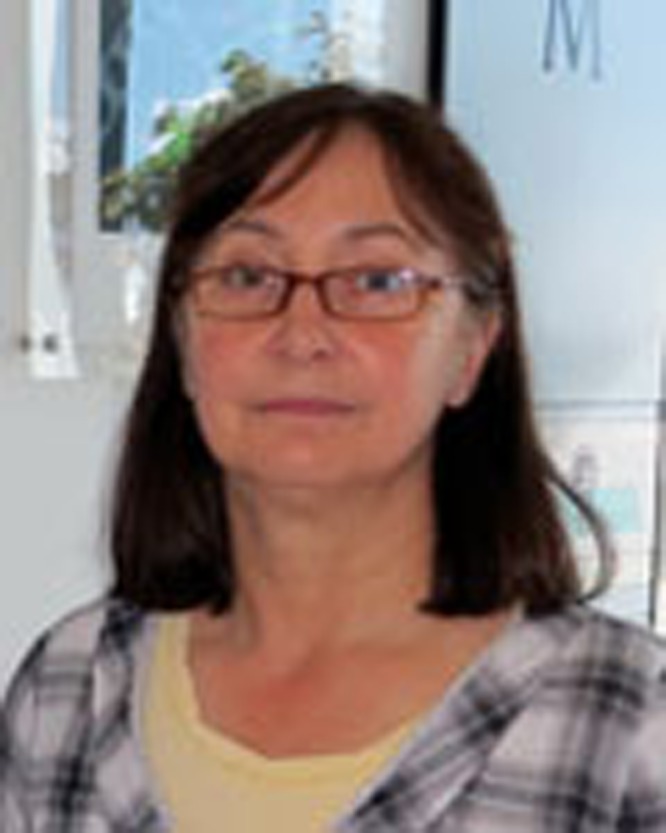
Lesley Clayton

**Figure illus3:**
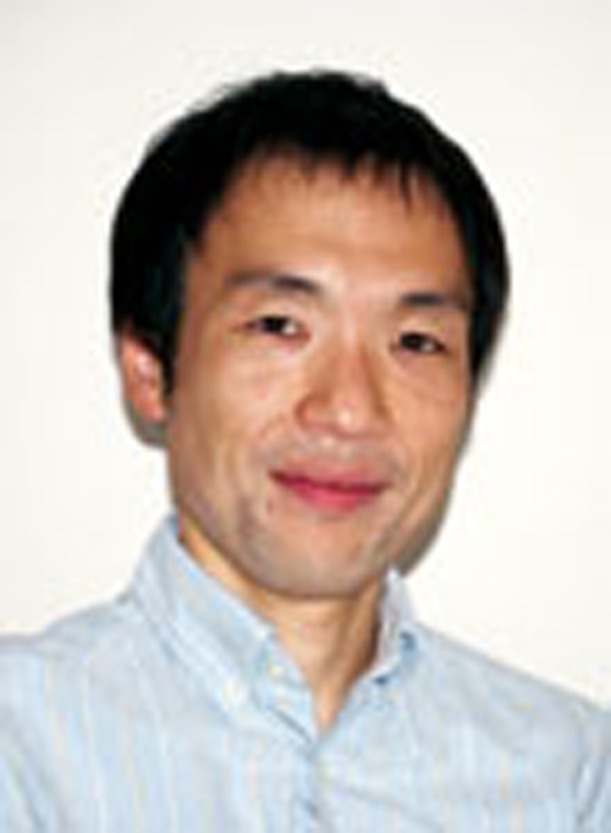
Toyoaki Natsume

## Glossary

CPC: chromosome passenger complex
CDE: centromere DNA element
DDK: Dbf4-dependent kinase
APC/C: anaphase-promoting complex/cyclosome
H3K9m: methylation of histone H3 at lysine 9
PP1/2A: phosphatase 1/2A
pre-RC: replicative complex

## Sidebar A | In need of answers

- How is the kinetochore–microtubule interaction stabilized when sister-kinetochore bi-orientation is established?
- How does robust pericentromeric cohesion promote sister-kinetochore bi-orientation?
- What is the advantage of early DNA replication of centromeric regions?
- How evolutionarily conserved is the centromere function of advancing the DNA replication timing of centromeric regions?
- How does DDK promote pericentromeric cohesion and advance DNA replication timing in budding and fission yeast?
- How have DDK and Shugoshin each acquired common functions in different contexts during evolution (in pericentromeric cohesion and replication timing)?
